# LOCAL INJECTION OF HUMAN DENTAL PULP STEM CELLS FOR TREATMENT OF JUVENILE AVASCULAR NECROSIS OF THE FEMORAL HEAD: PRELIMINARY RESULTS IN IMMATURE PIGS

**DOI:** 10.1590/1413-785220243201e283445

**Published:** 2025-04-07

**Authors:** LUIZ RENATO AGRIZZI DE ANGELI, GUSTAVO BISPO DOS SANTOS, JOSÉ RICARDO MUNIZ FERREIRA, BÁRBARA LÍVIA CORRÊA SERAFIM, THIAGO ZAQUEU LIMA, LUIZ GUILHERME CERNAGLIA AURELIANO DE LIMA, DANIELA FRANCO BUENO, ROBERTO GUARNIERO

**Affiliations:** 1 Universidade de Sao Paulo, Faculdade de Medicina, Hospital das Clinicas HC-FMUSP, Departamento de Ortopedia e Traumatologia DOT, Sao Paulo, SP, Brazil.; 2 Universidade Federal do Espírito Santo, Vitória, ES, Brazil.; 3 Universidade Federal do Rio Grande do Norte, Natal, RN, Brazil.; 4 Faculdade Israelita de Ciências da Saúde Albert Einstein, São Paulo, SP, Brazil.

**Keywords:** Legg-Calvé-Perthes Disease. Ischemia. Models, Animal. Swine. Dental Pulp. Stem Cells. Biological Treatment, Doença De Legg-Calvé Perthes, Isquemia, Modelos Animais, Suínos, Polpa Dentária, Células-Tronco, Tratamento Biológico

## Abstract

**Introduction:**

Legg-Calvé-Perthes disease is a major cause of hip joint deformities in children. Currently, experimental research is directed at investigating biological therapies, including the use of human dental pulp stem cells (hDPSC), which have not yet been studied for this purpose in swine models. This study aimed to evaluate whether local injection of hDPSC induces bone mineralization in the proximal femoral epiphysis in an experimental model of avascular necrosis of the femoral head in immature pigs.

**Methods:**

Ten immature pigs underwent surgery to induce osteonecrosis of the proximal femoral epiphysis on the right side. In the intervention group (IG), hDPSC injections were performed immediately after osteonecrosis induction, and in the control group (CG), no additional procedure was performed. Left hips were used as controls. After 8 weeks, all animals were euthanized, and macroscopic, radiographic, and histological evaluations were performed.

**Results:**

Bone mineralization was greater in the right hips of the IG compared to the CG (p = 0.0356), with an average mineralization index increase of 77.78% after hDPSC injection. Radiographic evaluation of the epiphyseal index showed a greater collapse in the right IG hips compared to the right CG hips (p < 0.001) and macroscopic evaluation showed a higher chance of the femoral head being flat (p = 0,049).

**Conclusion:**

The injection of hDPSC into the proximal femoral epiphysis with induced osteonecrosis increases bone mineralization in immature pigs, but these treated hips show more deformity compared to the untreated hips. ***Level of Evidence IV, Case Series*** .

## RESUMO

**Introdução:**

A doença de Legg-Calvé-Perthes é uma das principais causas de deformidades do quadril na infância. Pesquisas atuais exploram terapias biológicas, incluindo células-tronco da polpa dentária humana (CTPDh), cujos efeitos ainda não foram estudados em modelos suínos. Este estudo avalia se injeções locais de CTPDh promovem a mineralização óssea em um modelo experimental de necrose avascular da cabeça femoral no porco imaturo.

**Métodos:**

Dez porcos imaturos foram submetidos à osteonecrose induzida cirurgicamente na epífise femoral proximal do lado direito. O grupo de intervenção (GI) recebeu injeções de CTPDh, enquanto o grupo controle (GC) não recebeu tratamento adicional. Os quadris esquerdos serviram como controles. Após oito semanas, todos os animais foram submetidos à eutanásia e foram realizadas avaliações macroscópicas, radiográficas e histológicas.

**Resultados:**

A mineralização óssea foi maior no GI do que no GC (p = 0,0356), com aumento da mineralização de 77,78% após a injeção de CTPDh. O GI apresentou maior colapso da cabeça femoral (p < 0,001) e maior chance de a cabeça apresentar um formato plano (p = 0,049).

**Conclusão:**

As injeções de CTPDh aumentam a mineralização óssea nos quadris de porcos imaturos submetidos a osteonecrose isquêmica, mas levam a maior deformidade da cabeça femoral. **Nível de evidência IV, Série de Casos.**

## INTRODUCTION

Idiopathic avascular necrosis of the proximal femoral epiphysis configures a major cause of permanent hip joint deformities in children^1^ ^)-(^ ^3^. Clinical practice has found that Legg-Calvé-Perthes Disease (LCPD), which can affect patients aged from two to 14 years, occurs more often in boys. It is considered the most common form of pediatric patients’ femoral head osteonecrosis, showing an overall annual prevalence from 5.1 to 16.9 per 100,000^4^ ^)-(^ ^6^.

Treatment involves reducing the deformity of the femoral head and minimizing its complications^7^. Numerous forms of treatment have been proposed for LCPD^7^. However, the few cases to produce randomized clinical trials entails the scarce concrete evidence about the best treatment for the disease. Experimental studies in animal models have been carried out since the 1970s to try to find answers on the approach to the disease^8^ ^)-(^ ^10^. Several studies with animal models have shown important points regarding treatment options, such as non-weight bearing in affected limbs^1^ and biologic therapies to increase bone mineralization and decrease necrotic femoral head deformity^11^ ^)-(^ ^17^.

Human dental pulp stem cells (hDPSC) have an excellent potential for osteogenic differentiation^18^ ^)-(^ ^24^. However, the use of hDPSC to treat hip osteonecrosis is yet to be studied in immature swine models. Research in mature sheep has shown faster bone regeneration after locally implanting hDPSC in osteonecrosis-induced proximal femoral epiphyses^24^. Due to the feasibility of acquiring this type of biological material, which can be cultivated from a sample of temporary teeth from children, its study may offer an effective treatment for patients with LCPD in the future.

The primary objective of this pilot study was to evaluate whether locally injecting hDPSC induces bone mineralization in the proximal femoral epiphyses in an experimental model of avascular necrosis of the femoral head in immature pigs.

## METHODS

This comparative experimental clinical trial was approved by the Scientific Committee of the Institute of Orthopedics and Traumatology at Hospital das Clínicas at the School of Medicine at Universidade de São Paulo (IOT-HCFMUSP) under protocol IOT 1149 and by the Ethics Committee on the Use of Animals (CEUA) at FMUSP under protocol no. 141/15. The animals were kept at the Vivarium of the School of Medicine at Universidade de São Paulo (a suitable place to maintain the species) with a local caretaker. Cleaning, adequate disposal of waste following local sanitary standards, and suitable water and feed were offered to the animals.

## PRODUCTION OF THERMOSENSITIVE HYDROGEL

For this study, a bioabsorbable injectable bone substitute with bioactive properties was developed, which was composed of chitosan-xanthan-methylcellulose polymers and calcium phosphate granules in their hydroxyapatite phase.

First, the base production of the hydrogel was carried out in a mass ratio of 1:1 of chitosan and xanthan. The chitosan solution at 1% w/v (Sigma-Aldrich, 83% deacetylation) was prepared in a solution composed of 2% v/v lactic acid (Merck) dissolved in ultrapure water (Milli-Q Direct Q 8/16 System) and homogenized in a mechanical agitator (Tecnal, nautical impeller) at a 1000-rpm rotation. In addition to the chitosan-xanthan polyelectrolyte complex, the methylcellulose polymer was used at a concentration of 10%. The manufacturer (Sigma-Aldrich) and the literature state that the sol-gel transition occurs from 30 to 34 ºC for such concentration. The following steps were performed to add the methylcellulose: dispersion of the methylcellulose powder in 1/3 of the volume of solvent heated to 80 ºC; addition of another 1/3 of the volume of solvent at a temperature from 2 to 8 ºC in agitation; and addition of the remaining 1/3 of solvent to adjust the concentration and liquid behavior at low temperatures.

The calcium phosphate granules were obtained after synthesizing hydroxyapatite by a chemical precipitation in aqueous medium^25^ ^),(^ ^26^. The precipitated dust was then filtered with filter paper (Qualy, J.Prolab - 80 grams, 18.5 cm) in a vacuum system (DVP, model ZA 60S) at 400 mbar. The retained powder was washed with ultrapure water to remove its potassium ions until a pH of seven was obtained in the filtered liquid. The obtained powder was dried at 70 ºC for 12 hours to produce the hydroxyapatite granules. The powder was macerated by a grail and agate pistil and transferred to a particle size sieve from 150 to 300 μm (A bronzinox, 100 mesh and 5” x 2” stainless steel frame).

The final step to produce the chitosan-xanthan-methylcellulose hydrogel with hydroxyapatite particles consisted of mixing the products described above with a spatula at a temperature below 8 ºC until complete homogenization.

## ADVANCED THERAPY PRODUCT

To deliver the advanced therapy product, the cells were associated to the hydrogel in two stages:

- **Cell Preparation:** HDPSC (DPSC - PT5025, LONZA) were thawed and resuspended in 1× DPBS (Thermo Fisher Scientific). Cells were counted by Countess™ equipment, and a concentration of 1.0 x 10⁶ cells was centrifuged at 300 × g for six minutes. The supernatant was discarded, and the cell pellet was resuspended in 600 μL of 0.9% sodium chloride saline solution.
- **Hydrogel Cell Association:** A volume of 1.4mL of the sterilized hydrogel was homogenized with 600 μL of cells resuspended in a saline solution. The final 2 mL of the mixture were aspirated into a 5-mL syringe, ensuring an even distribution of the cells in the hydrogel. This process facilitates the manipulation of the material for extrusion and promotes an environment conducive to cell adherence and proliferation that are essential for bone regeneration.

## CELL VIABILITY TESTING

Triplicate samples were seeded in a 12-well culture plate for the viability test based on the MTT assay^27^ ^),(^ ^28^ (3-[4,5-dimethyl-thiazole-2-yl]-2,5-diphenyltetrazolium bromide) (Sigma-Aldrich, St. Louis, MO) in the groups referring to two samples of the hydrogel. After adding hDPSC to the hydrogel samples, they were cultured for 48 hours for cell adherence in the hydrogel with the basal culture medium (DMEM F12 + 15% fetal bovine serum). After this period, the samples were washed with 3 ml of PBS to remove the excess basal medium, then 500μl of 0.05mg/ml of MTT were added to the culture wells. All plates were properly wrapped in aluminum foil to keep them away from light for four hours in an incubator (Thermo Fisher Scientific,Waltham, Massachusetts, USA) at 37°C with 5% CO^2^. After incubation, 500μl of DMSO (Dimethyl sulfoxide; (CH3)2SO - Sigma-Aldrich, St. Louis, MO) were placed in each cultured well, and all plates were stirred in a horizontal stirrer for five minutes to homogenize the solution. Then, the contents were transferred to a 96-well reading plate and read in a spectrophotometer (TECAN, infinity 200 PRO, Switzerland) with a reading in a 570nm absorbance filter. The values were processed on Magellan 3. The absorbance of the solubilized product is directly proportional to the number of viable cells^29^ ^),(^ ^30^.

## STUDY DESIGN

Overall, 10 immature pigs aged from 70 to 165 days and weighing from 8 to 13 kg were used. Avascular necrosis of the right proximal femoral epiphysis was surgically induced in all animals. Their left hips were used as controls. The protocol to induce avascular necrosis was based on previously described models^31^ ^)-(^ ^36^.

**Figure 1:**
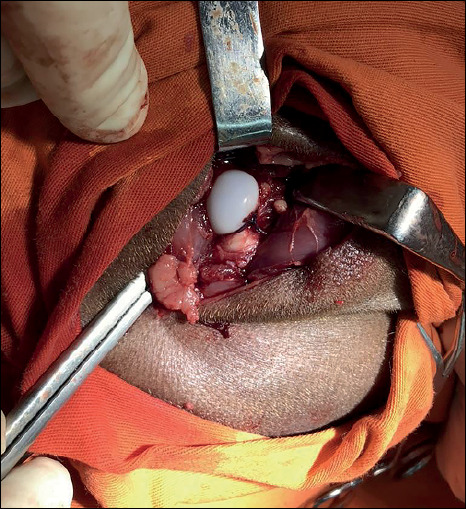
Photograph of the surgical procedure showing the intracapsular ligatures in the femoral neck with absorbable sutures after the ligamentum teres was sectioned.

The surgical procedure to induce necrosis consisted of performing two intracapsular ligatures around the neck of the right femur of the animals with absorbable Vicryl 2-0 sutures (Ethicon INc., Somerville, NJ) and the section of the ligamentum teres (Figure 1). The procedure aims to interrupt blood flow to the proximal femoral epiphysis. . After necrosis was inducted, hip reduction and wound closure in a standard fashion were performed in layers in the control group. In the intervention group, after necrosis induction, the hDPSC were injected into the proximal femoral epiphysis.

**Figure 2:**
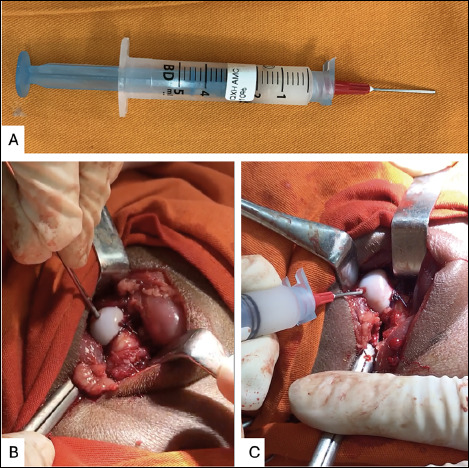
Photographs of the surgical procedure showing the direct injection of hDPSC into the proximal femoral epiphysis. A: syringe containing hDPSC and hydrogel. B: perforation of the proximal femoral epiphysis with 1.5-mm Kirschner wire. C: Injection of hDPSC and hydrogel into the proximal femoral epiphysis, taking care to avoid leaking the material.

The injection of hDPSC into the proximal femoral epiphysis was performed directly (Figure 2). A 1.5-mm Kirschner wire was used to perforate the articular cartilage of the femoral head, pointing to the center of the epiphysis. After the articular cartilage is passed, using a 5-ml syringe with a 1.2x25-mm needle filled with hDPSC added to Hydrogel, 2ml of the solution containing 1 x 10^6^ cells were injected into the femoral head, taking care to avoid extravasated material into the joint. After the needle was removed from the femoral epiphysis, the hole was occluded with bone wax and the hip was reduced at the joint. The wound was closed in a standard fashion.

The animals in both groups were observed for eight weeks from the day of ischemia induction, when they were euthanized to evaluate the results. The animals were allowed full weight-bearing as tolerated.

## ANATOMOPATHOLOGICAL AND HISTOLOGICAL EVALUATION

The right and left hips of the animals were carefully dissected. The samples were macroscopically and microscopically examined. In the macroscopic examination, the sphericity of the femoral head was evaluated based on the Stulberg classification, modified by Huhnstock et al. ^(^ ^37^ (flat, ovoid, and spherical).

For the microscopic evaluation, 3-mm thick cuts were made on the femoral heads and necks with a saw in the coronal plane. Each sample was analyzed macroscopically and microscopically. Each sample was fixed in 10% formalin, soaked in paraffin, and cut into 6-μm slices. The sections were stained with hematoxylin-eosin and von-Kossa staining and examined under ordinary light microscopy. Histomorphometric evaluations were performed by a specialist in bone pathology who was blinded to the groups.

## RADIOGRAPHIC EVALUATION

All hips underwent radiographic evaluation. Radiographic examinations of the right and left hips were performed in anteroposterior projection. The exams were carried out shortly after the euthanasia. The hips of the animals were dissected, and radiographs were performed only in the proximal region of the femurs, avoiding image overlapping artifacts.

The degree of femoral epiphysis deformation was measured by the epiphyseal index, which is defined as the maximum height divided by the maximum diameter of the proximal femoral epiphysis on an anteroposterior radiograph^38^. Flattening of the femoral epiphysis tends to decrease its height and increase its diameter, reducing the epiphyseal index. Measurements were performed on the picture archiving and communication system software at IOT-HCFMUSP.

## STATISTICAL ANALYSIS

Analysis aimed to test the effect of induction of avascular necrosis in the femoral head of pigs and to evaluate the effect of treatment with hDPSC injection on the femoral epiphysis after necrosis. This purpose was addressed in an experimental plan designed to independently test the factors. Thus, the inference of the effect of osteonecrosis was based on the comparison between the right joint (induced to avascular osteonecrosis) and the contralateral side spared from the lesion in the same animal, considering that neither side received treatment with hDPSC. Given the paired nature of this comparison, the assumption of independence between the measurements of both sides in the same individual becomes implausible, and the respective correlation is represented by the longitudinal character of the statistical model. Thus, the inference of the possible effect of inducing avascular osteonecrosis was based on the representation of its relationship with the outcomes of interest by parametric modeling in the scope of the generalized mixed linear models, the distribution of which was assumed, in each situation, according to the nature of the response and the function of linking the median of the response to the linear combination of the defined predictors to optimize fit. In all cases, the mixed component of the model, as defined by carrying out the experiment, consisted of the random effect of the individual, represented by an independent intercept for each animal.

In turn, the analysis of the effect of treatment with hPDSC injection was restricted to the subset of right-sided joints induced by avascular osteonecrosis, and only some of them were treated with pluripotent cell implants. In this unpaired context, the manifestation of treatment under the necrotic limb is compared with the state of the joints that are also injured but from different animals. Thus, the relations between treatment and the outcomes of interest were represented by generalized linear models so that the distribution assumed for the response was consistent with its nature and data and the connection of the mean response to the linear combination of predictors met the need for quality of fit. In the face of ordinal answers, cumulative link models, as generalizations of logistic regression under the assumptions of proportional odds (i.e., the odds ratio for an increase in the ordinal scale ignores the level of that scale) and flexible thresholds were used.

In any case, all inference was initially guided by a previous exploratory analysis, indicating possible relation patterns to support plausible models. After adjusting the data, the models were selected and validated to support valid inferences. Naturally, the validation methods were defined according to the class of the model and availability of the developed and deployed tools. In the case of the generalized mixed linear models, data representation by the model was validated by diagnostic measures based on the residuals of each model. In turn, the validation of a cumulative link model was supported by the Akaike information criteria, Bayesian information criteria, and the Hessian conditional matrix. Due to the small sample size, the evaluation of the statistical significance of each coefficient of a model (thus, of the effect represented by it) was carried out by maximum likelihood ratio test given its favorable convergence properties. Moreover, the statistical significance of any effect (or association) was set at a significance level α = 0.05. Finally, all analyses, graphs, and tables were generated on RStudio.

## RESULTS

### Cell Viability Assay

The normalized results below include the absorbance values for the negative control. Culture on both hydrogel samples showed the loss of about half of the cells. The comparison between hydrogel types showed no difference in viability regardless of the number of cells (Table 1).

**Table 1:** Cell viability assay in two hydrogel samples. The samples evaluated showed no difference in cell viability. The T-test obtained the p values.

		MTT ViabilityTest				Triple Average	Standard Deviation	p
HYDROGEL I	Positive Control	Cells	0.352	0.346	0.3663	0.355	0.01	
		Cells + Hydrogel I - 10^5^ cels/ml*	0.1979	0.1591	0.1622	0.173	0.02	
		Cells + Hydrogel I - 10^4^ cels/ml **	0.1589	0.1579	0.1625	0.160	0.00	
	Negative Control	Hydrogel I	0.0741	0.0627	0.0612	0.066	0.01	
								
HYDROGEL II	Positive Control	Cells	0.3453	0.3655	0.3772	0.363	0.02	
		Cells + Hydrogel II - 10^5^ cels/ml*	0.18568	0.18339	0.1845	0.185	0.00	0.394*
		Cells + Hydrogel II - 10^4^ cels/ml **	0.14632	0.14752	0.1664	0.153	0.01	0.41**
	Negative Control	Hydrogel II	0.0453	0.0447	0.0445	0.045	0.00	

Source: Prepared by the authors.

### Mineralization

**Figure 3:**
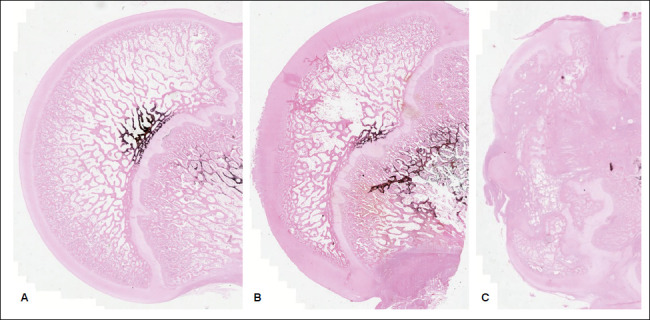
Microscopic images of histological sections stained by von Kossa staining (0.5x). A: Left hip of the control group. The femoral epiphysis has a normal trabecular distribution and spherical shape. B: Right hip from the control group. The femoral epiphysis has a heterogeneous and decreased trabecular distribution, loss of epiphyseal height, and a spherical shape. C: Right hip from the intervention group. The femoral epiphysis, despite greater collapse, shows greater trabecular bone density than the hips in Figures A and B.

**Figure 4:**
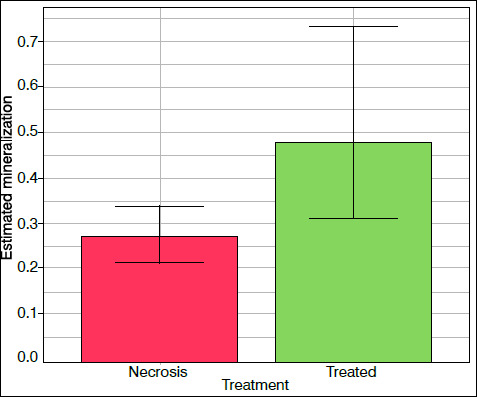
Effect of the treatment on the mineralization of the proximal femoral epiphysis. Columns indicate the values predicted by the model according to the treatment. In turn, the vertical bars illustrate the 95% confidence intervals for the respective estimated average value.

The statistical significance of the effect of treatment with hDPSC injection on necrotic femoral head mineralization followed the selection and validation of the statistical model (Table 2) (p = 0.0356). In this case, estimates suggest that the mean mineralization index increases by 77.78% after hDPSC injection in comparison to osteonecrosis-induced femoral heads without the application of the treatment (Figures 3 and 4).

**Table 2:** Effect of the treatment with hDPSC injection on the mineralization of the necrotic femoral head

Mixed linear model range with logarithmic binding for mineralization					
			Likelihood ratio		
	Estimate	Standard Error	Lambda	DF	p
Location / mean					
(Intercept)	1.30933	0.11889			
Treatment: treated x necrosis	0.575365	0.251607	4.414781	1	0.035629
Scale / Dispersion					
(Intercept)	2.649667	0.625142			
Treatment: treated x necrosis	1.24665	0.872256	1.908586	1	0.16712

Source: Prepared by the authors.

### Femoral Head Sphericity

**Figure 5:**
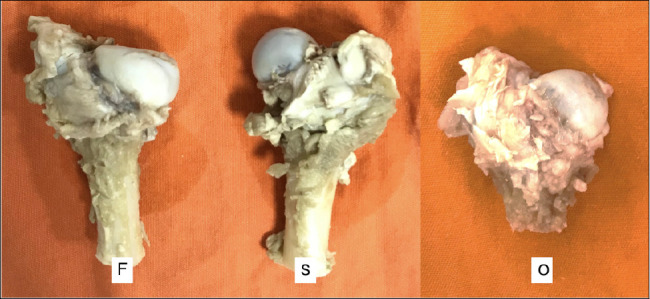
Macroscopic qualitative evaluation of the shape of the femoral head. F: Flat femoral head. S: Spherical femoral head. O: Ovoid femoral head.

In individuals who received no hDPSC treatment, 80% of the femoral heads spared from necrosis induction (controls) had a spherical appearance, and the remaining 20% evinced a single observation, whose femoral head was ovoid. In turn, the induction of necrosis in the ipsilateral limb of these same animals resulted in an ovoid appearance in 80% of cases and only a flat shape in the remaining ones. On the other hand, applying hDPSC seems to be associated with greater necrosis-induced deformation of the femoral heads since the proportion of cases with flat shape increased to 80%, followed by a reduced incidence of ovoid appearance in a single case. In other words, most femoral heads spared from manipulation had a spherical shape, whereas the limbs subjected to necrosis were predominantly ovoid when untreated with hDPSC injections. Treatment with hDPSC seems to have further affected the femoral head sphericity after necrosis since most cases showed a flat femoral head (Figure 5).

Treatment statistically and significantly affected the femoral head toward flatness (p = 0.0496). Implanting pluripotent cells may reduce the chance of the femoral head being ovoid by 93.75% when compared to heads that are only necrotic.

### Radiographic Epiphyseal Index

The inferential context confirmed the statistical significance of the effect of avascular osteonecrosis induction on the epiphyseal index (p = 0.0032). In this case, the epiphyseal index of the joints subjected to necrosis decreased, on average, by 7.1189 when compared to the limbs without avascular lesions. In addition to affecting the mean response, inducing osteonecrosis was also statistically significantly associated with an increased variability of radiographic epiphyseal index values (p = 0.0231).

**Figure 6:**
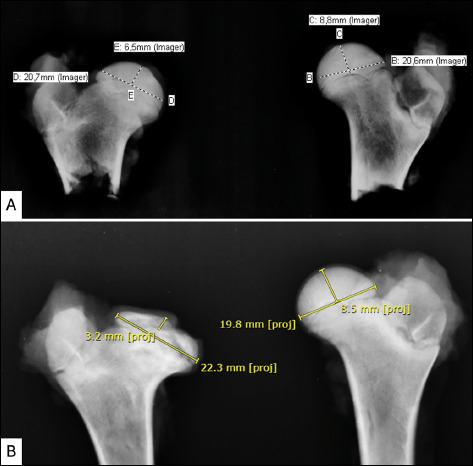
Example of the measurement of the radiographic epiphyseal index in the groups. A: Measurement image on a control group specimen. The epiphyseal index of the right hip equals 6.5/20.7 = 0.31, and that of the left hip, 8.8/20.6 = 0.42. B: measurement image on an intervention group specimen. The epiphyseal index of the right hip equals 3.2/22.3 = 0.14, and that of the left hip, 8.5/19.8 = 0.42.

**Figure 7:**
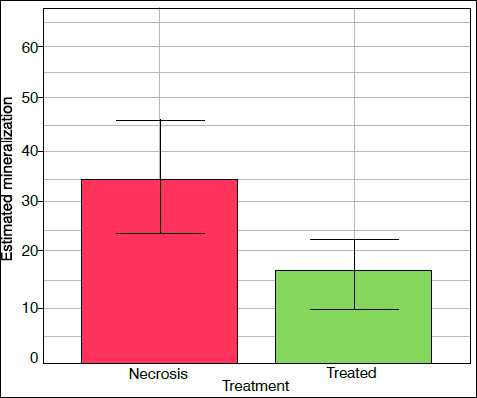
Effect of treatment on the epiphyseal index by comparing the right hips of the control (necrosis) and intervention groups (treated). Columns indicate the values the model predicted according to treatment. The vertical bars illustrate the 95% confidence intervals for the respective estimated average values.

This study also confirmed the statistical significance of the effect of hDPSC injection on the epiphyseal index of the limb previously induced to avascular osteonecrosis (p < 0.001). Thus, the robustness of the evidence can estimate that treatment reduces 49.22% of the mean epiphyseal index of a joint induced to avascular osteonecrosis in comparison with limbs with the same lesion without cell treatment (Figures 6 and 7).

### Treatment Safety

No specimen in this analyses showed heterotopic ossification or malignant transformation.

## DISCUSSION

The main motivation of this study involved testing the hypothesis that injecting hDPSC into the proximal femoral epiphysis of immature pigs induced to avascular necrosis would increase the bone mineralization of their epiphyses, generating greater support and preserving the morphology of the femoral head when compared to controls subjected to osteonecrosis alone. The primary outcome of this study (femoral epiphysis mineralization) showed a positive and statistically significant result that favored the main formulated hypothesis. However, the secondary outcome (maintenance of femoral head sphericity) was unfavorable to the tested hypothesis since the hips treated with hDPSC showed greater deformity than the hips only subjected to avascular necrosis.

Over the more than 100 years since the discovery of LCPD, numerous studies have been published on mechanical treatment options to the prevent deformation of the affected proximal femoral epiphysis based on the principle of femoral head containment^7^ ^),(^ ^39^. Surgical treatment options such as femoral osteotomies, acetabular osteotomies, and hip arthrodiastasis with external fixators configure the most common current options to treat the disease^7^ ^),(^ ^40^. Regarding conservative treatment, non-weight bearing has gained prominence in recent publications^41^ ^)-(^ ^43^. However, few studies have evaluated biological treatments for the disease in humans^44^ ^)-(^ ^46^. Herrera-Soto and Price have reported good results in the femoral epiphysis decompression in six adolescents with LCPD by a perforation with a cannulated drill and/or eight to 10-mm trephines and autologous bone grafting or synthetic bone substitutes^44^. The best results in this series occurred in patients who underwent operation during early fragmentation^47^. These authors published a review that recommended early decompression and shelf acetabular arthroplasty so these patients could obtain better results^46^. Sivakumar et al. ^(^ ^45^ published a report of two cases subjected to epiphyseal injection of 2mg zoledronic acid in patients diagnosed with LCPD. The authors claimed satisfactory results. No prospective or retrospective comparative studies in humans evaluating the efficacy of any biologic therapy for LCPD have been published to date.

Studies applying interventions in experimental animal models have shown good results, increasing the bone density of the femoral epiphysis and preserving femoral head sphericity ^11^ ^)-(^ ^13^ ^),(^ ^15^ ^)-(^ ^17^ ^),(^ ^24^ ^),(^ ^35^ ^),(^ ^48^ ^)-(^ ^50^. Cheng et al. ^(^ ^35^ ^)^ most closely resembles this study as they applied the interventions at the same time as they induced avascular necrosis. These authors concluded that the sucrose acetate isobutyrate offers an efficient means to carry bone morphogenetic protein 2 (BMP-2) to the proximal femoral epiphysis. They also showed that injecting recombinant human BMP-2 without bisphosphonate failed to prevent femoral epiphysis deformity. However, it maintained the shape of the femoral head well when injected with bisphosphonate.

Most experimental models that evaluated biological interventions applied them one or two weeks after osteonecrosis induction^11^ ^)-(^ ^13^ ^),(^ ^48^ ^)-(^ ^50^. Regarding studies that used BMP-2 as their main agent, Kim et al. ^(^ ^11^ experimented in 18 immature pigs, in which six animals received locally applied BMP-2; six, directly and locally applied BMP-2 associated with ibandronate; and six, no local biological therapy. The injections occurred from one to two weeks after osteonecrosis induction. Aruwajoye et al. ^(^ ^48^ also studied the injection of BMP-2 and ibandronate one week after osteonecrosis induction. The authors concluded that the treatment with BMP-2 or with it and ibandronate maintained the material properties of the femoral epiphysis similar to those of normal bone. Both studies concluded that associating BMP-2 with a bisphosphonate better preserve femoral head sphericity and bone mineralization. Our investigation did not use a resorptive agent, which may have contributed to femoral head sphericity loss in the treated group.

Aruwajoye et al. ^(^ ^13^, Wang et al. ^(^ ^49^, and Gong et al^12^ ^)^ tested transphyseal perforations as a biological treatment. Aruwajoye et al. ^(^ ^13^ compared multiple transphyseal perforations with single transphyseal tunneling in immature pigs subjected to classic induction of avascular osteonecrosis. The pigs underwent amputation of the right limb to simulate non-weight bearing. The interventions occurred one week after necrosis induction. Both interventions caused no growth defect in the proximal physis of the femur, and the multiple perforations stimulated greater bone formation than tunneling. Wang et al. ^(^ ^49^ associated a single transphyseal perforation with a 1.2-mm diameter metallic wire with the injection of BMP-2 and rabbit adipose tissue-derived stem cells in an experiment with 45 immature rabbits. The intervention occurred two weeks after osteonecrosis induction. The authors concluded that the transphyseal injection of BMP-2 and tissue-derived stem cells induced greater bone formation and less deformation of the femoral head than in the control group or the group that only underwent perforation without inoculations. Gong et al. ^(^ ^12^ performed three transphyseal perforations with 1.57-mm Kirschner wires in the left hips three weeks after medial necrosis induction. The authors found a higher percentage of hip revascularization in multiple perforations, which seems to produce no bony bars in the physis. However, the perforations neither prevented the collapse of the femoral head nor promoted bone reformation. Our study injected hDPSC directly into the femoral head, which may have contributed to greater deformation of the proximal femoral epiphysis than in the control group. Since Wang et al. and Gong et al. observed that the perforations were unable to prevent the collapse of the femoral epiphysis, a single perforation with a thin wire would very likely fail to increase bone formation in the epiphysis by itself. Therefore, the effect of the increased mineralization in our study can be attributed to hDPSC inoculation rather than to drilling.

Another biological treatment option refers to the systemic use of tocilizumab, a substance that inhibits interleukin 6, which is elevated in the synovial fluid of patients with LCPD and is produced by chondrocytes^14^. Kamiya et al. ^(^ ^50^ induced osteonecrosis in six-week-old immature rats by surgically cauterizing the four veins that supply the right proximal femoral epiphysis. The study compared them with a control group (for which that study found such veins but refrained from cauterizing them). The animals were treated with tocilizumab injections since the third day after osteonecrosis induction every week for six weeks (totaling six doses). The authors concluded that the drug increases bone volume in the injected epiphyses. Kuroyanagi et al. ^(^ ^17^ and Ren et al. ^(^ ^15^ have come to the same conclusion.

Our study has several limitations. Due to issues related to the supply of hDPSC and the inability to operate and maintain more than five animals concomitantly in our physical structure, this study ignored randomization. However, the described methodology of osteonecrosis induction and postoperative care were exactly the same between the groups, and the same team and the same surgeon performed all the experiments on all animals. Another limitation of this study refers to the absence of a third comparative group that would only undergo perforation of the femoral epiphysis without hDPSC injection. Although studies have evaluated the effect of single perforations in the femoral epiphysis with a control group - such as Wang et al. ^(^ ^49^ - to date, beneficial or harmful effects are yet to be shown on outcomes related to single fine-needle drilling in the femoral epiphysis. Studies such as Kim et al. ^(^ ^11^ considered a model to investigate the biological treatment of avascular necrosis of the hip in immature pigs also ignored a control group with isolated perforation. Thus, we believe that perforation failed to affect the results of our experiment. On the other hand, we stress that the association of directly perforating the femoral epiphysis without protecting loads may have influenced the decrease in the epiphyseal index in the intervention group.

Regarding the effect of hDPSC inoculation, this study was limited by the absence of cell labeling and evaluation of the results from immunohistochemical analyses. Wang et al. ^(^ ^49^ labeled adipose-derived stem cells in their experiment with bromodeoxyuridine. The authors concluded that the location of the cells gradually changed from the mesenchymal area of the femoral epiphysis to the trabecular bone from the fourth to the eighth week. They also observed that the intensity of the marking signal decreased up to the eighth week, probably suggesting that cell modulation on the cells of the own animals played an important role in increasing bone volume in their proximal femoral epiphyses. Therefore, we conclude that injecting hDPSC into the femoral epiphysis increased bone mineralization, although it is impossible to state whether the effect stemmed from the cellular modulation due to the implanted cells or from these cells directly increasing bone volume in the femoral head of the treated animals.

This study has several strengths. This pioneering study evaluated the use of hDPSC to treat induced osteonecrosis in a porcine animal model of Legg-Calvé-Perthes disease. Numerous studies have investigated biological therapies to increase bone mineralization and decrease the deformity of the femoral epiphysis due to avascular necrosis induction^11^ ^)-(^ ^13^ ^),(^ ^15^ ^)-(^ ^17^ ^),(^ ^24^ ^),(^ ^35^ ^),(^ ^48^ ^)-(^ ^50^. Adipose-derived stem cells, BMP-2, bisphosphonates, and systemic therapies such as tocilizumab injection showed benefits in models with pigs, rats, and rabbits. However, only one^24^ offered an option for a graft that induces bone formation that could be collected and cultured from temporary baby teeth, avoiding the need for a treatment with allografts or synthetic options that remain unavailable in several countries, such as BMP-2. The clinical feasibility of obtaining hDPSC in patients with LCPD is timely since patients who need intervention are usually aged from six to 10 years, the age at which the exchange of temporary and permanent teeth occurs naturally^7^. This would make the graft extraction method less invasive and morbid for patients. The clinical use of this type of graft to stimulate bone formation in patients diagnosed with cleft palate, for example, has been studied and its safety proven^51^ ^)-(^ ^53^. This study found neither side effects related to heterotopic ossification in the hip joint of the treated animals - as in Kim et al. ^(^ ^11^ by injecting BMP-2 and ibandronate - nor any evidence of cellular malignancy in the evaluated femoral epiphyses. The osteogenic potential of hDPSC has been extensively studied^18^, and this study adds another possible application of the use of this type of cell graft to possibly benefit patients with LCPD in the future.

Finally, injecting hDPSC into the proximal femoral epiphysis of immature pigs induced to avascular necrosis increases bone mineralization when compared to epiphyses induced only to avascular necrosis. Still, collapse and deformity of the femoral head occurred consistently in the treated epiphyses. Therefore, our future studies aim to associate transphyseal injections of hDPSC with the removal of limb support by transfemoral amputation to investigate whether it will be possible to preserve the sphericity of the epiphysis in association with increased bone mineralization. A future translational study is required to evaluate the possibility of testing treatment in humans in case of promising results regarding the maintenance of head sphericity.
